# Prognostic Factors and Optimal Surgical Management for Lumbar Spinal Canal Stenosis in Patients with Diffuse Idiopathic Skeletal Hyperostosis

**DOI:** 10.3390/jcm11144133

**Published:** 2022-07-16

**Authors:** Hideaki Nakajima, Kazuya Honjoh, Shuji Watanabe, Akihiko Matsumine

**Affiliations:** Department of Orthopaedics and Rehabilitation Medicine, Faculty of Medical Sciences, University of Fukui, 23-3 Matsuoka Shimoaizuki, Eiheiji-cho, Yoshida-gun, Fukui 910-1193, Japan; kazuya@u-fukui.ac.jp (K.H.); shujiw@u-fukui.ac.jp (S.W.); matsumin@u-fukui.ac.jp (A.M.)

**Keywords:** diffuse idiopathic skeletal hyperostosis, lumbar spinal canal stenosis, prognostic factors, surgical management, clinical outcome

## Abstract

Lumbar spinal canal stenosis (LSS) and diffuse idiopathic skeletal hyperostosis (DISH) tend to develop in the elderly, resulting in an increased need for lumbar surgery. However, DISH may be a risk factor for poor clinical outcomes following lumbar decompression surgery, especially in patients with DISH extending to the lumbar segment (L-DISH). This study aimed to identify the prognostic factors of LSS with L-DISH and propose an optimal surgical management approach to improve clinical outcomes. Of 934 patients who underwent lumbar decompression surgery, 145 patients (15.5%) had L-DISH. In multivariate linear regression analysis of the JOA score improvement rate, the presence of vacuum phenomenon at affected segments (estimate: −15.14) and distance between the caudal end of L-DISH and decompressed/fused segments (estimate: 7.05) were independent prognostic factors. In logistic regression analysis of the surgical procedure with JOA improvement rate > 25% in L-DISH patients with both negative prognostic factors, the odds ratios of split laminotomy and short-segment fusion were 0.64 and 0.21, respectively, with conventional laminotomy as the reference. Therefore, to achieve better clinical outcomes in cases with decompression at the caudal end of L-DISH, decompression surgery without fusion sparing the osteoligamentous structures at midline should be considered as the standard surgery.

## 1. Introduction

Diffuse idiopathic skeletal hyperostosis (DISH) has an unknown etiology, but is a non-inflammatory condition with features of calcification and ossification of connective tissues, especially in the spinal ligaments [1]. The prevalence of DISH was reported as 10.8% (male 22.0%, female 4.8%) in the cohort study in Japan, with a considerably higher prevalence in the elderly [2]. Previous studies showed that DISH appears in 38% and 49% in thoracic and thoracolumbar regions of cases, which is extended to the lumbar region in 13% [3,4]. On the other hand, lumbar spinal canal stenosis (LSS) is a common degenerative disease in the elderly. A cross-sectional study in Japan reported a prevalence of 9.3% for symptomatic LSS [5], with a higher prevalence in elderly patients aged 70 to 79 years [6]. Considering the predominance of the older age group in both diseases, DISH and LSS are not uncommon in clinical settings in today’s aging society.

Furthermore, the presence of DISH has been considered to be a risk factor for lumbar posterior decompression surgery with and without fusion for LSS [7,8,9,10]. In particular, the association with higher revision rates was indicated in patients with DISH extending to the lumbar segment (L-DISH) [11]. A previous report suggested that the cause of revision surgery in L-DISH patients who underwent posterior decompression surgery without fusion was the appearance of intervertebral instability at the decompressed segment after surgery [12]. If intervertebral instability is the cause of revision surgery, then the indication of spinal fusion is considered. However, poor surgical outcomes of fusion surgery have also been reported [8,13], and it is not clear which surgical strategy is appropriate as an index surgery for these patients to achieve better clinical outcomes. To prevent revision surgery, including residual symptoms such as low back pain and neurological symptoms, the preoperative risk factors other than the presence of L-DISH and the pathomechanisms for this condition need to be understood. However, there is limited information on preoperative prognostic factors and surgical strategies for avoiding revision surgery for LSS patients with L-DISH.

The aim of this study was to identify the predictors of poor surgical outcomes in LSS patients with L-DISH based on the Japanese Orthopaedic Association (JOA) score improvement rate at follow-up and to establish an approach for optimal surgical management to achieve better clinical outcomes with significant improvement.

## 2. Materials and Methods

### 2.1. Study Population

A total of 934 LSS patients underwent lumbar posterior decompression surgery without fusion or 1- or 2-level short-segment posterior lumbar interbody fusion (PLIF) between 2005 and 2019, and were followed up for >2 years. Patients who had undergone previous spinal surgery and those aged <50 years were excluded from the study. The Resnick criteria were used to diagnose DISH on lateral lumbar spine radiographs below T10 as follows: (1) ossification/calcification along the anterior aspect of at least 4 contiguous vertebrae, (2) a somewhat preserved intervertebral disc height, and (3) no sacroiliac joint erosion [1]. In addition, the Mata scoring system was used to evaluate contiguous ossification [14], and the study only included cases with complete bridging of the disc space (grade 3). Cases were grouped into those in which DISH reached the lumbar region (L-DISH) and all others (non-L-DISH).

### 2.2. Clinical Outcomes and Radiological Measurements

The JOA scoring system before and at follow-up, including those who underwent revision surgery within 2 years after surgery, was used to assess the clinical status. JOA score improvement rate was calculated using the following formula: (JOA score at follow-up—preoperative JOA score) × 100/(29—preoperative JOA score). Cases with a JOA score improvement rate of ≤25% were defined as a poor clinical outcome. The rate of revision surgery at the same segment and/or an adjacent segment was determined. Clinical data including age, gender, body mass index (BMI), and surgical procedures were obtained from medical charts. In L-DISH cases, preoperative plain radiography, computed tomography (CT), and magnetic resonance imaging (MRI) were used to examine if an intradiscal vacuum (appearance of an intervertebral radiolucent area caused by disc degeneration) or vertebral bone marrow edema (defined as an area with diffuse high and low intensity on T2- and T1-weighted images, respectively (Modic type I change)) was present at the decompression level [15]. Lumbar lordosis (LL), pelvic incidence (PI), and PI-LL were assessed by preoperative lumbopelvic radiography.

### 2.3. Surgical Procedure

We changed the surgical procedure of lumbar decompression from conventional laminotomy to lumbar spinous process-splitting (split) laminotomy in 2015 because of the advantages of a less invasive procedure for lumbar paraspinal muscles [16]. In conventional laminotomy, the paraspinal muscles were detached from the spinous process, followed by partial laminectomy between the cephalad and inferior lamina. In split laminotomy, the cephalad spinous process was longitudinally split with the attached bilateral paraspinal muscles to divide the structure into halves from the lamina base. Following decompression, the two halves were sutured together with the supra- and interspinous ligaments. In short-segment PLIF, bilateral total facetectomy with a conventional open midline approach was performed. Following disc material removal and vertebral endplate preparation, two cages were inserted into each intervertebral space filled with the resected local autologous bone.

### 2.4. Statistical Analysis

Data are presented as the median [interquartile range]. Mann-Whitney U-test or chi-square test were used to compare the categorical variables, with *p* < 0.05 considered significant. Factors that were significant in univariate analysis and other factors from the literature were included in multivariate regression analysis. The estimate, odds ratio, and 95% confidence interval (CI) were calculated to identify independent predictors of JOA score improvement. To assess the inter- and intraobserver reliabilities, intraclass correlation coefficients (ICCs) were calculated. EZR (Saitama Medical Center, Jichi Medical University, Saitama, Japan) [17], which is a graphical user interface for R (The R foundation for Statistical Computing, Vienna, Austria) were used for these analyses.

## 3. Results

### 3.1. Clinical and Radiological Features of Patients with L-DISH

Table 1 summarizes the patient data with and without L-DISH. The mean follow-up period was 4.8 ± 2.1 years. Of 934 patients who underwent lumbar decompression surgery, 145 patients (15.5%) were diagnosed with L-DISH. There were a significantly higher percentage of male patients (86.9% vs. 43.1%) and a larger number of older patients at the time of surgery in the L-DISH group. The L-DISH group had a significantly lower median postoperative JOA score and rate of JOA improvement at follow-up compared to the non-L-DISH group, despite the preoperative JOA score not differing significantly between the two groups. The rate of revision surgery was also significantly higher in the L-DISH group (9.0% vs. 4.4%, *p* = 0.0050).

Table 2 shows the imaging findings of patients with L-DISH. In around half of these patients, a vacuum phenomenon and/or vertebral bone marrow edema were observed at the decompressed/fused segments. In addition, more than half of the cases involved lumbar decompression surgery at the caudal end of L-DISH or one distance from the segment adjacent to L-DISH.

### 3.2. Prognostic Factors in L-DISH Patients Based on the JOA Improvement Rate

A comparison of L-DISH patients with and without JOA improvement rate > 25% at the final follow-up is shown in Table 3. There were significant differences in the caudal end of L-DISH, vacuum phenomenon at affected segments, Modic change at affected segments, and distance between the caudal end of L-DISH and decompressed/fused segments between patients with and without JOA improvement rate > 25%.

Multivariate linear regression analysis of the JOA score improvement rate was performed to identify independent prognostic factors in L-DISH patients (Table 4). The presence of vacuum phenomenon at affected segments (estimate: −15.14) and distance between the caudal end of L-DISH and decompressed/fused segments (estimate: 7.05) were identified as independent prognostic factors of the JOA score improvement rate in L-DISH patients. No significant differences were shown in the patient background, caudal end of L-DISH (upper/lower), PI minus LL, Modic change at affected segments, and surgical procedure.

### 3.3. Effect of the Surgical Procedure on the Postoperative Improvement of L-DISH Patients with Vacuum Phenomenon at a Lower Segment and One Distance from the Segment Adjacent to L-DISH

To clarify the surgical strategy for the affected segments with vacuum phenomenon at a lower segment or one distance from the caudal end of L-DISH, logistic regression analysis of the surgical procedure with JOA improvement rate > 25% was performed. Of the 145 patients, 44 patients (30.3%) were included in the analysis. Although no significant statistical differences were observed, the odds ratios of split laminotomy and short-segment fusion were 0.64 and 0.21, respectively, with conventional laminotomy as the reference (Table 5).

## 4. Discussion

The high mechanical stress on segments free of ossification due to a longer lever arm at the distal end of L-DISH can induce disc degeneration or ligamentum flavum hypertrophy, which has been associated with a requirement for surgery for subsequent development of LSS [7,8,9,10,11]. As LSS and DISH tend to develop in the elderly, the demand for lumbar surgery is expected to become more common with the aging of society. In the current study, 86.9% of the patients were male; thus, the presence of DISH should be particularly considered in elderly male patients. Surgery for LSS patients with DISH is more likely to be required compared to that in non-DISH cases, but DISH may also be an independent risk factor for revision surgery following lumbar decompression surgery [8,11,12,13]. In our study, the revision rate was significantly higher in patients with L-DISH compared with those without L-DISH (9.0% vs. 4.4%). Therefore, the aim of this study was to identify the prognostic factors in surgically-treated LSS patients with DISH and propose an optimal surgical procedure to achieve better clinical outcomes.

The current study showed that the presence of vacuum phenomenon and distance of affected segments from L-DISH were independently and significantly associated with the JOA score improvement rate. In a previous study, CT findings revealed that a vacuum phenomenon was associated with the severity of disc degeneration and LSS, with an incidence rate of 5.6–9.2% at lumbar segments [18]. In our study, this rate was considerably high (44.8%) among L-DISH patients, which suggests that lumbar vertebrae experience a significantly higher mechanical load in L-DISH. Similar to a vacuum phenomenon at affected segments, a vertebral bone edema (Modic type I change) on MRI may be associated with the acceleration of intervertebral disc degeneration and low back pain [19,20,21]. A previous study suggested that preoperative findings of a Modic type I change on MRI could be an important predictor of the early progression of intervertebral disc degeneration after decompression surgery [22]. In the current study, Modic type I change was more common in L-DISH patients with a JOA score improvement rate ≤ 25% than in those with a JOA score improvement rate > 25%. However, in multivariate linear regression analysis, the presence of Modic type I change was not a significant independent prognostic factor of the JOA score improvement rate. These results suggest that careful consideration should be given to L-DISH patients with vacuum phenomenon at the affected level rather than those with Modic type I change. A vacuum phenomenon is one of the imaging findings indicating intervertebral mobility and may be a reason to consider fusion surgery for the affected intervertebral spine [23]. Furthermore, assuming that pain and disabilities result from intervertebral instability, fusion surgery is a logical procedure with well documented good clinical outcomes. However, our results showed that the odds ratio of short-segment fusion surgery for JOA score improvement was rather low; thus, in any case, short-segment fusion surgery should not be recommended for L-DISH patients as the standard surgery. The therapeutic goal of fusion surgery is to achieve intervertebral bony fusion and to eliminate pathological motion. A previous study demonstrated that DISH was associated with the occurrence of pseudoarthrosis and/or ASD after short-segment PLIF, with rates of 25.6% among patients with DISH and 6.5% among those without DISH [8]. Short-segment PLIF is not recommended as standard surgery, especially for a lower segment and one distant from the segment adjacent to L-DISH, because of high rates of postoperative symptoms related to ASD (48.1%) and pseudoarthrosis (29.6%) [12]. The rate of ASD is significantly lower in another study of patients who underwent single-segment PLIF, which reported that 19% of patients had radiological ASD at 2 years [24]. On the other hand, the rate of pseudoarthrosis (29.6%) among patients with L-DISH is significantly higher compared with that in previous studies, which reported that the incidence of pseudoarthrosis after PLIF was less than 4% [25,26]. Short-segment fusion surgery at close to the lower end of L-DISH was considered to have rather poor clinical outcomes due to the relatively high rate of pseudoarthrosis and ASD against further mechanical stress to the fused segment [13]. Considering the poor clinical outcomes of short-segment fusion, indications for longer fusion surgery for L-DISH patients might be considered. However, caution should be taken when performing long fusion surgery because it is highly invasive for elderly patients, with a high rate of postoperative complications such as proximal junctional failure [27] and postoperative daily activity limitation [28,29], as observed in cases of spinopelvic fixation for patients with spinal deformity.

Similarly to fusion surgery, decompression surgery without fusion has been reported to have a high revision rate [9]. A higher revision rate has also been found for L-DISH compared with DISH without ossification in the lumbar region [11]. DISH is also an independent risk factor for revision surgery after decompression without fusion, with revision rates of 19% (vs. 6.9% for patients without DISH) and 9.8% (vs. 4.9%) in follow-up periods of 5 and 3 years, respectively [6,7,9]. The revision rates among L-DISH patients are considerably high, considering the results of a long-term analysis for revision rates at the same spinal level were 0.6% at 1 year, 1.7% at 5 years, and 2.7% at 10 years after decompression without fusion [30]. Another study reported that the rate of revision required at a lower segment adjacent to L-DISH due to the appearance of intervertebral instability was significantly higher after split laminotomy compared to that after conventional laminotomy (37.5% vs. 7.7%), especially for an affected segment between L2 and L4 at the lower end of L-DISH [12]. Split laminotomy is known as a less invasive surgical procedure that reduces postoperative wound pain and paraspinal muscle atrophy compared to conventional laminotomy, which is widely used as the standard surgical technique for LSS. In patients without L-DISH, the incidence of postoperative developed spinal instability due to splitting the posterior osteoligamentous structures, which is one of the most important structural elements of the spine, has not been described [31,32]. A previous study suggested a significantly lower occurrence of ASD in split laminotomy than those in conventional laminotomy, although the quality of evidence is low [33]. However, ossification or calcification of the supraspinous and interspinous ligaments is common in cases of DISH. Although split laminotomy is a less invasive surgical procedure that can minimize damage to the paraspinal muscles, for patients with L-DISH, the invasion of posterior anatomical structures such as supraspinous and interspinous ligaments might lead to even higher mechanical stress on the affected segment [16]. Based on the findings of the current study, we recommend use of a surgical method that can preserve the posterior osteoligamentous structures at the midline instead of lumbar split laminotomy and/or short-segment fusion.

There are several limitations in this study. First, it had a retrospective and single-center design including a small number of patients. Second, the whole range of DISH was not assessed because plain radiographs of the thoracic spine above T10 were not assessed in all cases of this study. Third, further investigation is needed to define the optimal surgical procedure, including minimally invasive surgery such as endoscopic surgery [34] and longer fusion surgery. Despite these limitations, the results of the study provide key insights and management on surgical strategy that should improve outcomes for LSS patients with L-DISH.

## 5. Conclusions

The presence of a vacuum phenomenon and a short distance from the caudal end of L-DISH of the affected segment were negative preoperative prognostic factors in LSS patients with L-DISH. To achieve better clinical outcomes and prevention of revision surgery for these patients, short-segment fusion surgery is not recommended as standard surgery. It is advisable to perform decompression surgery without fusion, and a surgical procedure conserving spinous process and supra/interspinous ligaments at the midline should be selected, instead of lumbar spinous process-splitting laminotomy.

## Figures and Tables

**Table 1:** Differences in background and clinical data between patients with and without L-DISH.

	L-DISH	Non-L-DISH	*p* Value
Patients, *n* (%)	145 (15.5%)	789 (84.5%)	
Male	126 (86.9%)	340 (43.1%)	<0.001 *
Female	19 (13.1%)	449 (56.9%)	
Age at operation, median {IQR}, years	73.0 {68.0, 78.0}	71.0 {65.0, 76.0}	0.032 *
BMI, median {IQR}	24.50 {22.5, 27.0}	24.0 {22.0, 25.5}	0.36
Preoperative JOA score, median {IQR}	15.0 {13.0, 17.0}	15.0 {13.0, 18.0}	0.49
Postoperative JOA score, median {IQR}	22.0 {19.0, 24.0}	23.00 {20.8, 24.0}	0.0070 *
JOA improvement rate, median {IQR}	46.7 {30.8, 60.0}	50.00 {40.0, 63.6}	0.026 *
Surgical Procedure, *n*			
Conventional laminotomy	85	360	
Split laminotomy	32	191	
Short-segment fusion	28	238	
Revision Surgery, *n* (%)	13 (9.0%)	27 (4.4%)	0.0050 *

IQR: interquartile range; BMI, body mass index; JOA, Japanese Orthopaedic Association. * *p* < 0.05.

**Table 2:** Imaging findings of patients with L-DISH.

Caudal End of L-DISH, *n* (%)	
L1	69 (47.6%)
L2	39 (26.9%)
L3	21 (14.5%)
L4	12 (8.3%)
L5	4 (2.8%)
Lumbar lordosis (LL), median {IQR}, degree	31.8 {24.3, 38.7}
Pelvic incidence (PI) minus LL, median {IQR}, degree	19.3 {10.8, 27.0}
Vacuum phenomenon at affected segments, *n* (%)	65 (44.8%)
Vertebral bone marrow edema at affected segments, *n* (%)	70 (48.3%)
decompressed/fused segments: distance from L-DISH, *n* (%)	
At lower segment adjacent to L-DISH	45 (31.0%)
At 1 segment lower	32 (22.1%)
At 2 or above segment lower	68 (46.9%)

IQR: interquartile range.

**Table 3:** Comparison of L-DISH patients with and without JOA improvement rate > 25%.

	JOA Improvement Rate > 25%	JOA Improvement Rate ≤ 25%	*p* Value
Patients, *n* (%)	116	29	
Male	100 (86.2%)	26 (89.7%)	0.85
Female	16 (13.8%)	3 (10.3%)	
Age at operation, median {IQR}, years	72.00 {68.00, 77.75}	75.00 {71.50, 78.00}	0.45
BMI, median {IQR}	24.50 {22.50, 26.78}	24.60 {22.70, 27.95}	0.60
Preoperative JOA score, median {IQR}	15.00 {13.25, 17.75}	14.00 {13.00, 17.00}	0.55
Caudal end of L-DISH, *n* (%)			
L1	63 (54.3%)	6 (20.7%)	0.0017 *
L2	31 (26.7%)	10 (34.5%)	
L3	14 (12.1%)	7 (24.1%)	
L4	5 (4.3%)	6 (20.7%)	
L5	3 (2.6%)	0 (0%)	
PI minus LL (degree), median {IQR}	18.05 {10.35, 26.35}	20.10 {14.15, 28.95}	0.44
Vacuum phenomenon, *n* (%)	39 (33.6%)	25 (86.2%)	<0.001 *
Modic change, *n* (%)	47 (40.5%)	22 (75.9%)	0.0014 *
Distance from L-DISH, *n* (%)			
At lower segment adjacent to L-DISH	28 (24.1%)	16 (55.2%)	0.0017 *
At 1 segment lower	26 (22.4%)	7 (24.1%)	
At 2 or above segment lower	62 (53.4%)	6 (20.7%)	
Surgical Procedure, *n* (%)			
Conventional laminotomy	73 (62.9%)	12 (41.4%)	0.086
Split laminotomy	24 (20.7%)	8 (27.6%)	
Short-segment fusion	19 (16.4%)	9 (31.0%)	

JOA, Japanese Orthopaedic Association; IQR, interquartile range; BMI, body mass index; PI, pelvic incidence; LL, lumbar lordosis. * *p* < 0.05.

**Table 4:** Multivariate linear regression analysis of the JOA score improvement rate.

Variables	Estimate	95% CI	*p* Value
Patient background			
Age (per 1 year)	−0.11	−0.61–0.39	0.66
Sex (female as reference)	−1.81	−14.16–10.54	0.77
BMI (per 1 kg/m^2^)	−0.56	−1.71–0.59	0.34
Imaging findings			
Caudal end of L-DISH (upper/lower) (upper (L1 and L2) as reference)	−5.10	−17.64–7.45	0.42
PI minus LL (per 1 degree)	−0.12	−0.46–0.22	0.49
Presence of vacuum phenomenon at affected segments	−15.14	−24.51–−5.78	0.0018 *
Presence of Modic change at affected segments	−3.72	−12.71–5.27	0.41
Distance from L-DISH (adjacent to L-DISH as reference)	7.05	0.65–13.45	0.031 *
Surgical procedures (conventional laminotomy as reference)			
Split laminotomy	−3.58	−13.75–6.58	0.28
Short-segment fusion	−5.74	−16.22–4.75	0.49

JOA: Japanese Orthopaedic Association; CI, confidence interval; BMI, body mass index; PI, pelvic incidence; LL, lumbar lordosis. * *p* < 0.05.

**Table 5:** Logistic regression analysis of the surgical procedure with JOA improvement score > 25% for patients with vacuum phenomenon at a lower segment and one distance from the segment adjacent to L-DISH.

Surgical Procedure	Odds Ratio	95% CI	*p* Value
Conventional laminotomy	1		
Split laminotomy	0.64	0.13–3.03	0.57
Short-segment fusion	0.21	0.033–1.36	0.10

CI, confidence interval.

## Data Availability

The data presented in this study are available on request from the corresponding author and subject to the ethical approvals in place and materials transfer agreements.
